# Effect of Al on the Isothermal Oxidation Behavior of a Ti_70_Zr_20_Ta_10_ Shape Memory Alloy at 900 °C

**DOI:** 10.3390/ma19122589

**Published:** 2026-06-16

**Authors:** Xiaolong Pang, Ailian Liu, Lei Liang, Jiawen Xu, Zhaiping Yang, Cundi Han

**Affiliations:** 1 School of Materials Science and Engineering, Heilongjiang University of Science and Technology, Harbin 150022, China; pangxiaolong@usth.edu.cn (X.P.); lianglei0513@sina.com (L.L.); xujiawen@usth.edu.cn (J.X.); yangzhaipingxp@163.com (Z.Y.); 2 College of Science, Heilongjiang University of Science and Technology, Harbin 150022, China; hancundi@usth.edu.cn

**Keywords:** Ti-Zr-Ta high-temperature shape memory alloys, Al, isothermal oxidation, microstructure

## Abstract

**Highlights:**

- Ti_70_Zr_20_${\mathrm{Ta}}_{10-x}$Al_x_ (x = 0, 0.5, 1, 3 at.%) alloys were prepared by replacing Ta with Al in $Ti_{70}Zr_{20}Ta_{10}$ shape memory alloy.
- The isothermal oxidation kinetics of Ti_70_Zr_20_${\mathrm{Ta}}_{10-x}$Al_x_ alloys strictly follow a parabolic law at 900 °C.
- The oxide films formed on the Ti_70_Zr_20_Ta_10_ alloy surface are mainly composed of TiO_2_, Ta_2_O_5_ and (Ti,Zr)O_2_, with inherent pores and cracks in the unmodified alloy.
- The Ti_70_Zr_20_Ta_9_Al_1_ alloy exhibits the optimal high-temperature oxidation resistance among Ti_70_Zr_20_${\mathrm{Ta}}_{10-x}$Al_x_ (x = 0, 0.5, 1, 3 at.%) alloys.

**Abstract:**

$Ti_{70}Zr_{20}Ta_{10}$ alloy is a β-titanium based shape memory alloy with a high martensitic transformation temperature and large recoverable strain. It is thought to be to develop into a new generation of high-performance high-temperature shape memory alloy materials. By partially replacing the Ta element in the $Ti_{70}Zr_{20}Ta_{10}$ alloy with Al, Ti-Zr-Ta-Al alloys with different Al contents were prepared. In this study, isothermal oxidation tests at 900 °C were conducted on Ti-Zr-Ta-Al alloys with different Al contents to investigate the effect of Al content on the high-temperature oxidation behavior of the $Ti_{70}Zr_{20}Ta_{10}$ alloy. The results show that the isothermal oxidation kinetics curves of $Ti_{70}Zr_{20}Ta_{10-x}Al_{x}$ (x = 0, 0.5, 1, 3 at.%) at 900 °C all follow a parabolic law. The oxide films formed on the alloy surface are mainly composed of $\mathrm{Ti}O_{2}$, $Ta_{2}O_{5}$ and $\left(\mathrm{Ti},\mathrm{Zr}\right)O_{2}$. However, the surface of the oxide films is relatively rough. The films are not dense and there are pores and cracks, leading to spallation during the oxidation process. After the addition of Al, the high-temperature oxidation resistance of the Ti-Zr-Ta alloy is improved. When the Al content is 1 at.%, $Ti_{70}Zr_{20}Ta_{9}Al_{1}$ exhibits the best high-temperature oxidation resistance.

## 1. Introduction

Shape memory alloys (SMAs), due to their excellent mechanical properties, high shape recovery ability, pseudoelasticity and biocompatibility, have become one of the most important alloys in both scientific and commercial fields. Because of these unique properties, these alloys are applied in aerospace, marine, automotive, medical, orthodontic, and other fields. Although research on many properties of these alloys has been rapidly growing, understanding of certain phenomena remains incomplete. In recent years, the high-temperature oxidation performance of alloys has become a subject of more in-depth study. Oxidation at high temperatures can change the chemical composition of the alloy surface. At the same time, high-temperature oxidation may also impair the shape memory effect (SME) in this region. Furthermore, SMAs need to be processed in vacuum and inert gas environments during multiple high-temperature treatment stages. As a result, the manufacturing of SMAs entails significantly increased costs.

Currently, research on the high-temperature oxidation behavior of Ti-Ni-based alloys is relatively extensive [1,2,3,4,5,6,7,8]. Zhao et al. [9] studied the effect of adding up to 7 at.% Nb on the oxidation of Ti-Ni-Nb alloys at 600 °C and 800 °C. They found that the oxidation resistance of the alloy increased proportionally with the Nb content, indicating the beneficial effect of Nb. When Ni-Ti alloys are oxidized at high temperatures, the primary oxide formed is $\mathrm{Ti}O_{2}$ [9], which is mainly influenced by thermodynamic and kinetic factors. In other Ti-based alloys that form $\mathrm{Ti}O_{2}$, the addition of Nb generally enhances the oxidation resistance of the alloy. Some reports indicate that oxidation resistance is proportional to the Nb content. However, when the Nb content reaches a certain level, the oxidation rate of the alloy decreases. Moreover, the surface and internal structure of the alloy change significantly after oxidation at different temperatures. Especially after annealing at 850 °C, partially spherical Nb is distributed in the Ni-Ti matrix and a network distribution of Ni-Ti-Nb appears. This may affect the mechanical properties and shape memory effect of the alloy. Kim et al. [10] systematically studied the high-temperature oxidation behavior of Ti-49Ni-12Hf high-temperature shape memory alloys. They investigated the effect of Hf addition on the oxidation performance of the alloy in dry air at 800 °C to 1000 °C. The study showed that, compared to conventional Ti-Ni alloys, the addition of Hf significantly improves the high-temperature oxidation resistance of the alloy. This is mainly due to the formation of a Hf-rich oxide layer beneath the outer oxide layer, which effectively hinders the diffusion of Ti ions. Furthermore, the research indicated that the growth rate of the Hf-rich oxide layer is significantly higher than that of other oxide layers. The oxidation kinetics of TiNiHf alloys exhibit complex behavior related to temperature and oxidation time. In the initial stage of oxidation, the alloy follows a parabolic rate law. While after a certain period, the oxidation rate gradually shifts to near-linear growth over time.

Ti-based high-temperature shape memory alloys, particularly alloy systems such as Ti-Zr, Ti-Nb and Ti-Mo, can be rolled with high deformation at room temperature owing to their excellent workability. The maximum deformation can reach 90% of the initial thickness. This greatly simplifies the industrial production process for fabricating these alloys into desired shapes and dimensions, such as fine wires, thin sheets and other profiles. In addition, these alloys possess high phase transformation temperatures, enabling them to retain their unique shape memory properties over a wider temperature range. Ti-Zr alloys undergo martensitic transformation within the single-phase region. Compared with other low-temperature shape memory alloys such as the Ni-Ti alloys series, Ti-Zr alloys exhibit higher transformation temperatures and are suitable for a broader temperature range [11], especially under elevated temperature environments. Furthermore, the martensitic transformation temperature and shape memory characteristics can be tailored by adjusting composition ratio and heat treatment process of alloys, allowing these alloys to meet diverse application requirements. Zirconium is a β-phase stabilizing element. With increasing Zr content in alloys, the lattice constant and unit cell volume increase. The micro-substructure of the martensitic phase is twinned [12]. Compared with Ti-Zr alloys, the shape memory effect and martensitic transformation stability of Ti-Zr-Ta alloys can be further improved by adding the third element tantalum. The addition of aluminum can enhance the properties of alloys. First, the incorporation of Al improves the corrosion resistance of titanium alloys. Second, Al reduces the deformation temperature of titanium alloys and increases their strength and hardness. In addition, the addition of Al enhances the thermal stability of titanium alloys [13].

Researches show that the addition of Al could improve the mechanical properties of Ti-Nb alloys and mainly plays the roles of solid solution strengthening and fine grain strengthening. Moreover, the phase transformation temperatures of Ti-Nb alloys are decreased, and the superelasticity and shape memory effect are both improved with the addition of Al [14,15]. When Al is added to Ti-Zr alloys, the microhardness and tensile strength of Ti-Zr alloys both increase. Zhang et al. [16] reported that as the Al content increased from 1% to 4%, the As temperature decreased from 534 K to 490 K. The Ti-20Zr-10Nb-3Al alloy exhibits the maximum shape memory recovery strain of 3.2%. After annealing at 873 K and 973 K, the elongation of the Ti-20Zr-10Nb-3Al alloy is nearly 18%. In our previous work, when no more than 3 at.% Al was added to the $Ti_{70}Zr_{20}Ta_{10}$ alloy, Al was solid-dissolved in $\alpha^{\prime \prime }$ martensite. The phase transformation temperatures increase with the increase in Al content, and the mechanical properties are improved simultaneously. When the Al content is 1 at.%, the tensile strength and elongation of the $Ti_{70}Zr_{20}Ta_{9}Al_{1}$ alloy are 1188 MPa and 13.95%, respectively, while those of the $Ti_{70}Zr_{20}Ta_{10}$ alloy are only 970 MPa and 9.5% [17].

Since Ti-based high-temperature shape memory alloys must undergo hot working processes such as hot forging, hot rolling, hot extrusion, and hot drawing to be fabricated from cast ingots into wires or plates. During the hot working process, a protective atmosphere is generally not adopted. The alloy surface exposed to air is inevitably oxidized. Moreover, as a high-temperature shape memory alloy, high-temperature oxidation is an unavoidable issue during service. Therefore, it is essential to investigate the high-temperature oxidation behaviors of shape memory alloys. Titanium alloys typically have a rational hot working temperature range of 750 °C–1000 °C, and Ti-Ta and Ti-Zr-Ta alloys have a hot working temperature of 900 °C [18,19,20,21,22]. To the best of the authors’ knowledge, there are few studies on the 900 °C isothermal oxidation response of Ti-Zr-Ta alloys. Hence, the isothermal oxidation behaviors of the $Ti_{70}Zr_{20}Ta_{10}$ alloy at 900 °C and the effect of Al addition on its isothermal oxidation performance were investigated in the present work.

## 2. Experiment

The equipment used for melting and preparing $Ti_{70}Zr_{20}Ta_{10-x}Al_{x}$ alloys is an arc melting furnace. The Al content in $Ti_{70}Zr_{20}Ta_{10-x}Al_{x}$ alloys is 0 at.%, 0.5 at.%, 1 at.% and 3 at.%, respectively. Before undergoing isothermal oxidation experiments, theses samples are subjected to solution treatment. The isothermal oxidation experiment of $Ti_{70}Zr_{20}Ta_{10-x}Al_{x}$ alloys in this article were conducted in a KSL-1100X resistance furnace (Hefei Kejing Materials Co., Ltd., Hefei, China). To ensure the accuracy of the experiment, the length, width and thickness of these samples were measured three times and averaged. Before being subjected to constant temperature oxidation, the sample is weighed in an alumina crucible. The total weight of the sample and crucible was measured using an electronic balance with a sensitivity of 0.1 mg. The constant temperature oxidation method used in this article is intermittent oxidation. These samples were placed in a muffle furnace and subjected to constant temperature oxidation for 50 h. The quality of the samples was measured at 1 h, 3 h, 5 h, 8 h, 10 h, 15 h, 20 h, 25 h, 30 h, 35 h, 40 h, 45 h, and 50 h of constant temperature oxidation. Due to the high oxidation rate of $Ti_{70}Zr_{20}Ta_{10-x}Al_{x}$ alloys in the early stage of oxidation, the state of these samples after 10 h of constant temperature oxidation needs to be understood. Firstly, the effect of Al on the oxidation resistance of $Ti_{70}Zr_{20}Ta_{10-x}Al_{x}$ alloys was analyzed using oxidation kinetics method. Secondly, the phase state, microscopic results and composition of the sample were analyzed using a Zeiss Axio lab.1 optical microscope (Carl Zeiss Microscopy GmbH, Göttingen, Germany), equipped with an energy dispersive spectrometer (EDS) (Bruker Nano GmbH, Berlin, Germany), a D/Max-2500 pc X-ray diffractometer (XRD) (Rigaku Corporation, Tokyo, Japan) and a Raman spectrometer (HORIBA, Ltd., Paris, France). Due to the weak adhesion between the oxide film formed by constant temperature oxidation of the sample and the substrate, the cross-section of the oxide film of the sample was analyzed by electroless nickel plating.

## 3. Results and Discussion

### 3.1. Kinetic Analysis of Isothermal Oxidation in $Ti_{70}Zr_{20}Ta_{10-x}Al_{x}$ Alloys

**Figure 1:**
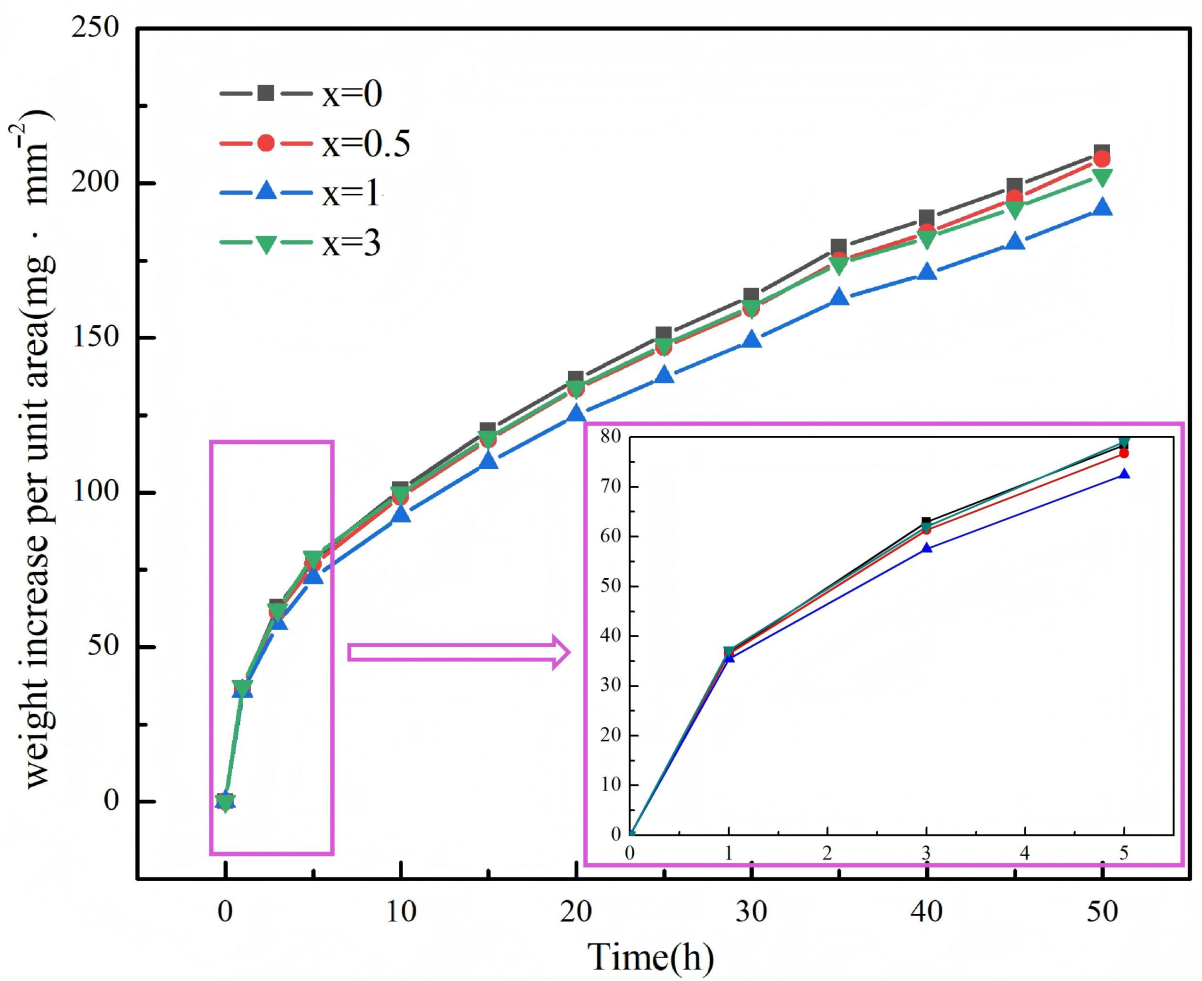
Kinetic curve of $Ti_{70}Zr_{20}Ta_{10-x}Al_{x}$ alloys oxidation at constant temperature at 900 °C.

**Figure 2:**
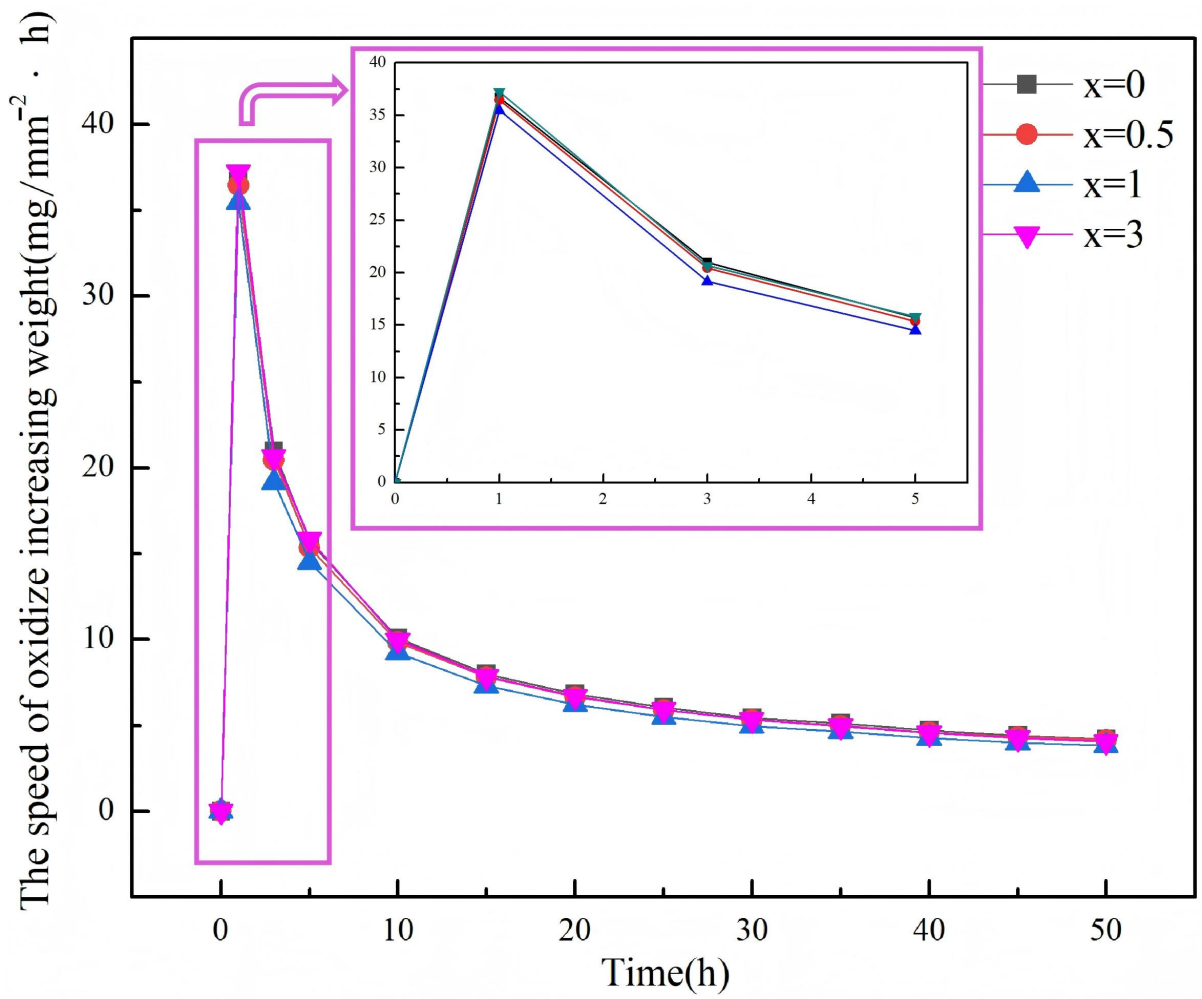
Effect of isothermal oxidation time on oxidation mass gain for $Ti_{70}Zr_{20}Ta_{10-x}Al_{x}$ alloy.

The kinetic curves of $Ti_{70}Zr_{20}Ta_{10-x}Al_{x}$ alloys with different content of Al at constant temperature for 50 h at 900 °C and the relationship between oxidation mass gain rate and time are shown in Figure 1 and Figure 2. It can be seen from subgraph in Figure 1 that when the isothermal oxidation time is 0–3 h, the mass gain per unit area of $Ti_{70}Zr_{20}Ta_{10-x}Al_{x}$ alloys increase rapidly and then decreases slightly within 3–5 h. As the oxidation time continues to increase, its mass gain per unit area continues to increase. When no Al was added, the $Ti_{70}Zr_{20}Ta_{10-x}Al_{x}$ ternary alloys gained the most mass per unit area. When the Al content did not exceed 1 at.%, the mass gain per unit area of $Ti_{70}Zr_{20}Ta_{10-x}Al_{x}$ alloys decreased with the increase in Al content. When the Al content exceeded 1 at.%, the weight gain per unit area of $Ti_{70}Zr_{20}Ta_{10-x}Al_{x}$ alloys increased with the increase in Al content. Therefore, when the Al content is 1 at.%, the mass gain per unit area of $Ti_{70}Zr_{20}Ta_{10-x}Al_{x}$ alloys is the smallest among the four alloys with different aluminum contents. The subgraph in Figure 2 shows that the oxidation and mass gain rate of the four alloys increases linearly from 0 h to 1 h. When alloys are oxidized in 1–3 h, the rate of oxidation mass gain decreases linearly. After that, as the oxidation time of alloys is prolonged, the rate of oxidation mass gain gradually decreases. Among the four test alloys, the ternary $Ti_{70}Zr_{20}Ta_{10-x}Al_{x}$ alloys without Al had the largest oxidation and mass gain rate. The oxidation and mass gain rate of the alloy with Al content of 1 at.% was the lowest.

When the $Ti_{70}Zr_{20}Ta_{10-x}Al_{x}$ alloys are high-temperature oxidized during the first 20 h, its mass gain per unit area occurs relatively rapidly. After 20 h of high-temperature oxidation, the rate of oxidation slows down. The oxidation mass gain rate of $Ti_{70}Zr_{20}Ta_{10-x}Al_{x}$ alloys is $209.78\;\mathrm{mg}/cm^{2}$. When the Al content is 0.5 at.%, the oxidation mass gain rate of the alloy is $207.76\;\mathrm{mg}/cm^{2}$. When the Al content is 1 at.%, the oxidation mass gain rate of the alloy is $191.61\;\mathrm{mg}/cm^{2}$. When the Al content is 3 at.%, the oxidation mass gain rate of the alloy is $202.55\;\mathrm{mg}/cm^{2}$. The data indicate that the addition of Al can enhance isothermal temperature oxidation resistance of the alloy. As the Al content continues to increase, the oxidation resistance of the alloy gradually deteriorates. However, compared to the $Ti_{70}Zr_{20}Ta_{10}$ alloy, the oxidation mass gain rate is reduced. Under the same high-temperature conditions, the amount of oxide formed on the alloy surface continues to increase with oxidation time. This oxidation process mainly undergoes two distinct stages. The first stage is the continuous growth stage of oxidation. The second stage is the steady-state stage of the oxidation reaction.

In the early stage of oxidation, the Ti, Ta, Zr and Al elements in the $Ti_{70}Zr_{20}Ta_{10-x}Al_{x}$ alloys all react with O to form oxide particles. Therefore, the oxidation rate of the $Ti_{70}Zr_{20}Ta_{10-x}Al_{x}$ alloys increases rapidly, leading to a significant mass gain in a short period of time. Particularly in the first 5 h, the oxidation mass gain curve is relatively steep. This indicates that the alloy has a high oxidation rate during this period. However, after the oxidation process continues for more than 30 h, oxidation curves of the alloy become more gradual. This indicates that although the mass of the alloy continues to increase due to oxidation, the rate of mass gain per unit time is significantly lower than before. At this point, the oxidation reaction of the alloy has entered a steady-state stage.

The reason for this change is that in the initial stage of oxidation, a large amount of oxygen is adsorbed on the surface of the alloy in molecular form and rapidly reacts with the metal atoms on the surface of the $Ti_{70}Zr_{20}Ta_{10-x}Al_{x}$ alloys [23]. The mass of the alloy increases rapidly due to oxidation. As the reaction progresses, a dense oxide layer gradually forms on the surface of the alloy. This oxide layer effectively prevents oxygen molecules from further penetrating and reacting with the alloy. Therefore, the oxidation rate gradually decreases and stabilizes. This steady-state stage indicates that the oxide layer formed on the surface provides protection and reduces further oxidation loss of the alloy. Therefore, this process offers a certain degree of oxidation resistance to the alloy. In summary, after experiencing the initial rapid oxidation stage, the formation of the surface oxide layer on the $Ti_{70}Zr_{20}Ta_{10-x}Al_{x}$ alloys promote the oxidation reaction to enter a relatively stable state. This process demonstrates the material’s adaptability and protective mechanism in a high-temperature oxidation environment [24].

The oxidation rate of the alloy is controlled by the diffusion between oxygen and the components in the alloy. It can be expressed as [25]:

(1) $${\left(\frac{\Delta m}{S}\right)}^{2}=K_{p}\cdot t,$$

where Δm represents the mass gain of the sample, *S* represents the sample area, *t* represents the oxidation time, and their units are mg, $mm^{2}$, and h, respectively. $K_{p}$ represents the oxidation rate.

**Figure 3:**
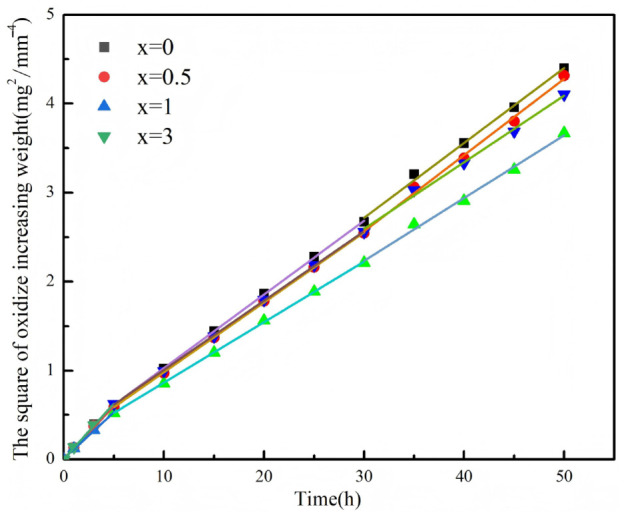
Isothermal oxidation ${\left(\Delta m/S\right)}^{2}$ versus *t* for $Ti_{70}Zr_{20}Ta_{10-x}Al_{x}$ alloys at 900 °C.

Figure 3 shows the relationship curves of ${\left(\Delta m/S\right)}^{2}$ versus time *t* for $Ti_{70}Zr_{20}Ta_{10-x}Al_{x}$ alloys during isothermal oxidation at 900 °C for 50 h. The slope obtained from the linear fit of this curve represents the oxidation rate constant $K_{p}$ of the alloy at each temperature. The specific values of $K_{p}$ are listed in Table 1. As seen in Figure 3, for all four alloys, ${\left(\Delta m/S\right)}^{2}$ versus *t* exhibits a linear relationship during the oxidation process. However, as the oxidation time of the alloy increases, the slopes of the lines change. This indicates that the $Ti_{70}Zr_{20}Ta_{10-x}Al_{x}$ alloys fully follow the parabolic law during isothermal oxidation. With the extension of time, the oxidation rate of the alloy gradually slows down and eventually stabilizes.

Table 1 shows the $K_{p}$ values of $Ti_{70}Zr_{20}Ta_{10-x}Al_{x}$ alloys oxidized at 900 °C for a constant time. As can be seen from the table, the ${\left(\Delta m/S\right)}^{2}$ and *t* curve of $Ti_{70}Zr_{20}Ta_{10-x}Al_{x}$ alloys oxidized at 900 °C for 50 h can be divided into three stages. The first stage is from 0 to 5 h, the second stage is from 5 to 30 h, and the third stage is from 30 to 50 h. In the first stage, as the Al content increases, the $K_{p}$ value initially decreases and then increases during the oxidation of the alloy. When the Al content is 1 at.%, the $K_{p}$ value of the $Ti_{70}Zr_{20}Ta_{9}Al_{1}$ alloy is the lowest and is $0.104\;mg^{2}\cdot mm^{-4}$. The trends of $K_{p}$ in the second and third stages are similar to those in the first stage, with the $Ti_{70}Zr_{20}Ta_{9}Al_{1}$ alloy having the lowest $K_{p}$ value.

In the initial stage of alloys oxidation, the oxidation rate is significantly higher compared to other stages. During the early phase of alloys oxidation, oxygen molecules first dissociate into atomic oxygen on the surface of the test alloy. These oxygen atoms subsequently adsorb onto the $Ti_{70}Zr_{20}Ta_{10-x}Al_{x}$ alloys surface and begin diffusing, adsorbing, or dissolving within the metal alloy lattice. When the interaction between oxygen atoms and the $Ti_{70}Zr_{20}Ta_{10-x}Al_{x}$ alloys reaches a saturation state in the lattice solubility, oxides start nucleating on the alloy surface and gradually grow. Eventually, a layer of oxide film forms on the alloy surface.

In the initial stage of alloys oxidation, the oxide film formed on the alloy surface is extremely thin. The interfacial reaction rate between the alloy and oxygen determines the initial growth rate of the oxide film on the alloy surface. As the oxidation process progresses, this oxide film gradually thickens and the oxidation reaction rate on the alloy surface slows down. At this point, the diffusion of oxygen atoms in the oxide film becomes more important. Because it determines the rate at which oxides on the alloy surface further grow and the final oxidation state.

During the isothermal oxidation process, the oxidation kinetics shifts from being interface reaction-controlled to diffusion-controlled. In this stage, the growth rate of the oxide film primarily depends on the diffusion capacity of oxygen atoms within the film. Consequently, as the thickness of the oxide films increases, its growth rate gradually decreases due to the limiting diffusion rate of oxygen atoms. The surface oxidation mass gain rate of the alloy decreases over time and tends to stabilize.

### 3.2. Phase Analysis of Oxide in $Ti_{70}Zr_{20}Ta_{10-x}Al_{x}$ Alloys

**Figure 4:**
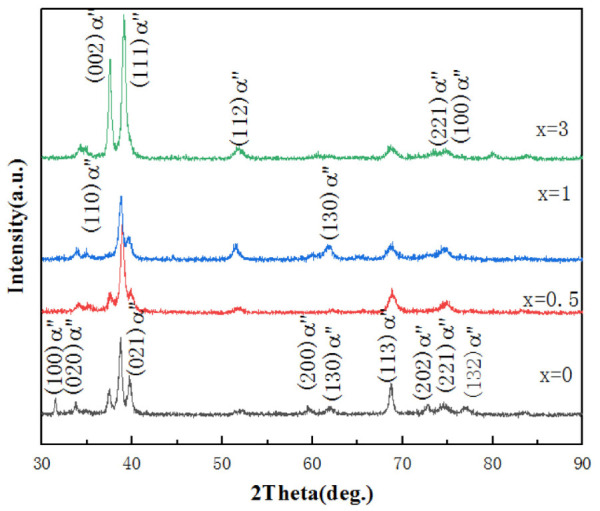
XRD of $Ti_{70}Zr_{20}Ta_{10-x}Al_{x}$ alloys at room temperature.

Figure 4 shows the room-temperature XRD of alloys with different Al contents under the solution-treated state. According to reference [17], the solution-treated $Ti_{70}Zr_{20}Ta_{10}$, $Ti_{70}Zr_{20}Ta_{9.5}Al_{0.5}$ and $Ti_{70}Zr_{20}Ta_{9}{\mathrm{Al}}_{1}$ alloys exhibit a single $\alpha^{\prime \prime }$ martensite phase at room temperature. By contrast, the solution-treated $Ti_{70}Zr_{20}Ta_{7}Al_{3}$ alloy consists of both $\alpha^{\prime \prime }$ martensite phase and $\alpha^{\prime }$ martensite phase at room temperature.

**Figure 5:**
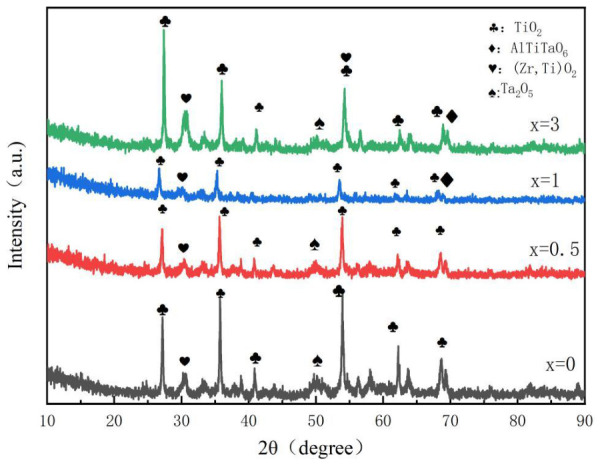
XRD curves of $Ti_{70}Zr_{20}Ta_{10-x}Al_{x}$ alloys oxidized at 900 °C for 10 h.

**Figure 6:**
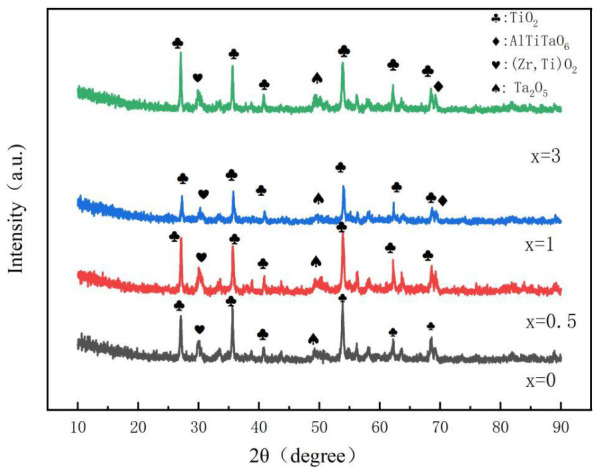
XRD curves of $Ti_{70}Zr_{20}Ta_{10-x}Al_{x}$ alloys oxidized at 900 °C for 50 h.

Figure 5 and Figure 6 show the XRD curves of $Ti_{70}Zr_{20}Ta_{10-x}Al_{x}$ (x = 0, 0.5, 1, 3 at.%) alloys after isothermal oxidation at 900 °C for 10 and 50 h, respectively. When the oxidation time of the alloy is 10 h, the oxide film of $Ti_{70}Zr_{20}Ta_{10}$ alloy is mainly composed of rutile $\mathrm{Ti}O_{2}$ (PDF card number: 21-1276), $Ta_{2}O_{5}$ phase (PDF card number: 54-0514), and $\left(\mathrm{Ti},\mathrm{Zr}\right)O_{2}$ (PDF card number: 35-0584) phase. After adding Al element, in addition to rutile $\mathrm{Ti}O_{2}$, $Ta_{2}O_{5}$, and $\left(\mathrm{Ti},\mathrm{Zr}\right)O_{2}$ phases, Al forms $\mathrm{AlTiTa}O_{6}$ (PDF card number: 32-0028) phase with Ti, Ta, and oxygen. The phase composition of the oxide film on $Ti_{70}Zr_{20}Ta_{10-x}Al_{x}$ alloys after 50 h of isothermal oxidation at 900 °C is the same as that after 10 h of oxidation. 

**Figure 7:**
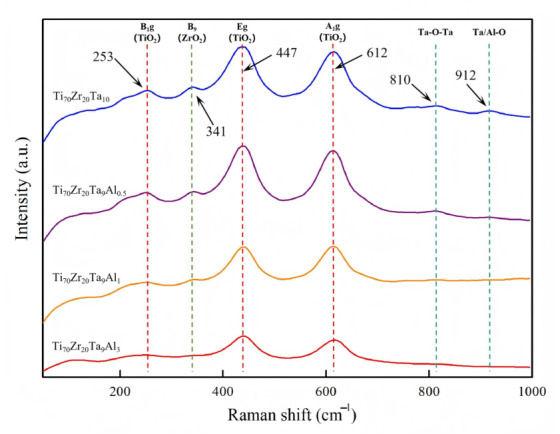
Raman curves of $Ti_{70}Zr_{20}Ta_{10-x}Al_{x}$ alloys oxidized at 900 °C for 50 h.

Figure 7 shows the Raman spectra of alloys with different Al contents. From this spectrum, it can be seen that the Ti-O, Zr-O, and Ta/Al-O Raman peaks exist on the surface of alloys. Therefore, these phases, such as $\mathrm{Ti}O_{2}$, $\mathrm{Zr}O_{2}$, and $Ta_{2}O_{5}$, may form on the surface of the $Ti_{70}Zr_{20}Ta_{10-x}Al_{x}$ alloys after oxidation. At the same time, with the increase in Al content, the intensities of the Ti-O, Zr-O, and Ta/Al-O Raman peaks gradually decrease. Therefore, the Al added to the $Ti_{70}Zr_{20}Ta_{10}$ alloy can inhibit the formation of Ti-O, Zr-O, and Ta/Al-O bonds.

### 3.3. Analysis of Surface and Cross-Sectional Morphology of Oxide Film

Before the isothermal oxidation test, the surfaces of all four alloys appeared silver white. When the alloy is subjected to isothermal oxidation at 900 °C, the color of the alloy surface gradually changes from silver white to earthy yellow. When $Ti_{70}Zr_{20}Ta_{10-x}Al_{x}$ alloys were subjected to isothermal oxidation at 900 °C for 10 h, the oxide film on the surface of the tested alloy cracked and peeled off. When the Al content is 1 at.%, the surface cracks of $Ti_{20}Zr_{20}Ta_{9}Al_{1}$ alloys are small. The oxide films on the surfaces of the other three alloys all showed coarse cracks. When the alloy was subjected to isothermal oxidation at 900 °C for 50 h, the surface oxide film of the four alloys cracked severely. After 10 h of isothermal oxidation, the surface of $Ti_{70}Zr_{20}Ta_{10}$ turns soil yellow. As the Al content increases, the color of the oxidized alloy surface gradually becomes lighter.

**Figure 8:**
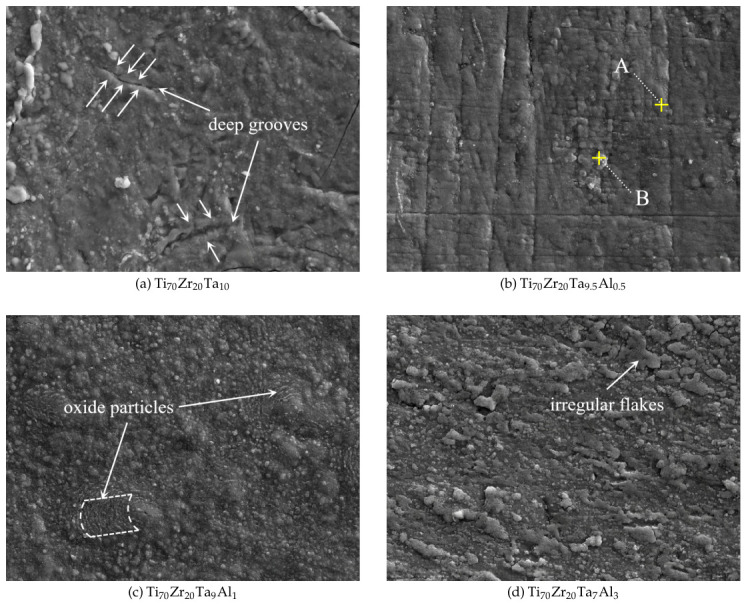
Surface morphology of $Ti_{70}Zr_{20}Ta_{10-x}Al_{x}$ alloys 900 °C after isothermal oxidation for 10 h.

Figure 8 shows the surface morphology of the oxide film on $Ti_{70}Zr_{20}Ta_{10-x}Al_{x}$ alloys after isothermal oxidation at 900 °C for 10 h. As shown in the figure, when the alloy is subjected to isothermal oxidation at 900 °C for 10 h, oxide particles of different sizes form on the surface of $Ti_{70}Zr_{20}Ta_{10}$ alloy. Moreover, there are cracks and pores in the oxide film formed on the surface of the alloy. Oxide nucleates and grows at the scratches on the surface of the alloy. When the Al content is 0.5 at.%, deep grooves form on the surface of the oxide film of the alloy. Therefore, the surface of the oxide film is not flat. The oxide particles formed on the surface of the alloy are mostly relatively small. At the same time, many spherical particles formed by the accumulation of oxide particles exist on the surface of the alloy. It indicates that the Al element can promote a denser oxide film on the surface of the $Ti_{70}Zr_{20}Ta_{10-x}Al_{x}$ alloys and make the oxide particles finer and more uniform. When the Al content is 1 at.%, the oxide film is relatively dense and the oxide particles on the alloy surface are relatively uniform. Some of the oxide particles are distributed in an arc shape. When the Al content is 3 at.%, the surface of the oxide film becomes rougher. Some of the oxide film has peeled off. Some of the oxide particles that have not fallen off are coarse. Some of the oxides accumulate together to form irregular flakes.

Table 2 shows the qualitative element content analysis of micro-area fixed-point in Figure 8b. According to the table, the content of O element in both micro-regions is about twice that of Ti element. Therefore, in the A and B micro-regions, the oxide is mainly $\mathrm{Ti}O_{2}$ phase.

**Figure 9:**
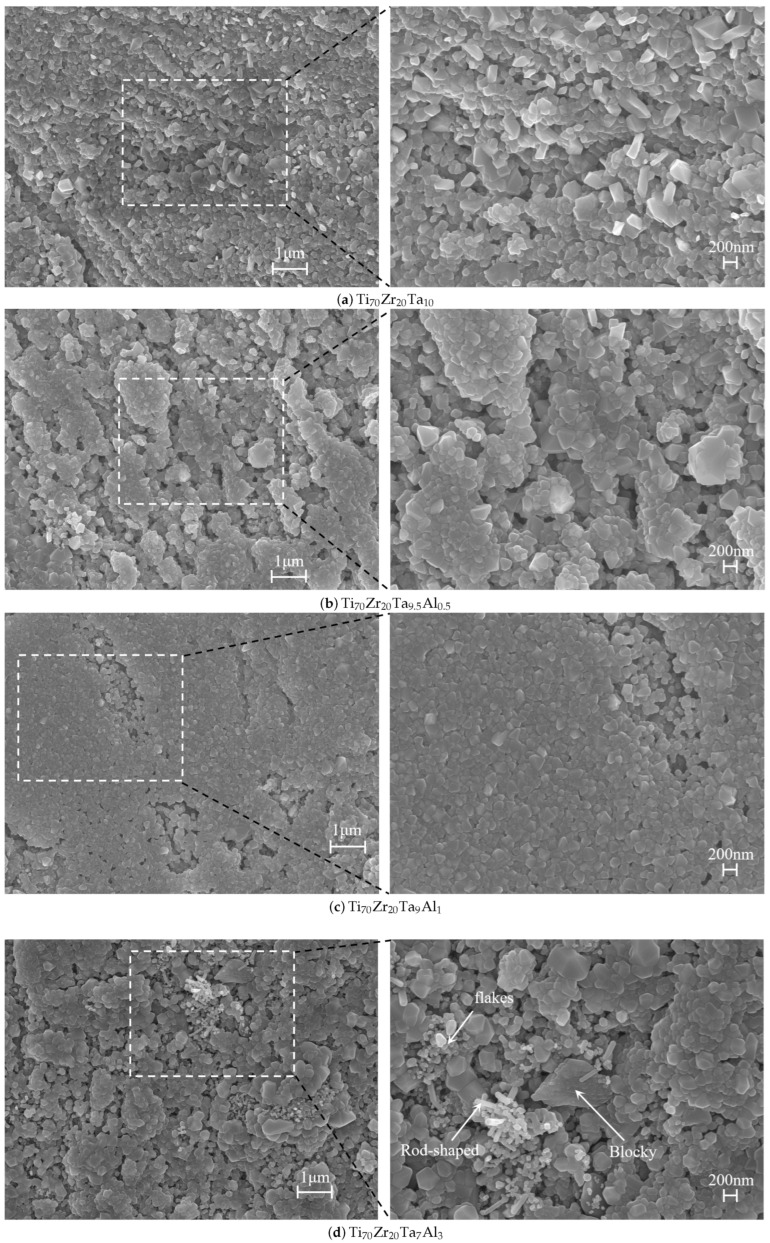
Surface morphology of $Ti_{70}Zr_{20}Ta_{10-x}Al_{x}$ alloys 900 °C after isothermal oxidation for 50 h.

Figure 9 shows the surface morphology of $Ti_{70}Zr_{20}Ta_{10-x}Al_{x}$ alloy after 50 h of constant temperature oxidation at 900 °C. As shown in the figure, after 50 h of oxidation, the surface oxide particles of the alloy gradually increase. There are a large number of oxide particles on the surface of $Ti_{70}Zr_{20}Ta_{10}$ alloy. But there are many gaps between these particles. Therefore, the oxide film formed on the surface of the alloy without added Al is not dense. When the aluminum content is 0.5 at.%, the oxide particles on the surface of the alloy aggregate and form blocks. However, there are still a large number of gaps between the formed block oxides. When the aluminum content is 1 at.%, the oxide particles on the surface of the alloy aggregate and connect together. As shown in Figure 9c, the oxide film formed on the surface of the alloy is dense. As shown in Figure 9d, when the aluminum content is 3 at.%, there are a large number of rod-shaped, block shaped, and sheet-like particles on the surface of the alloy. These particles are not interconnected. Therefore, when the aluminum content is 3 at.%, the oxide film on the surface of the alloy is loose.

**Figure 10:**
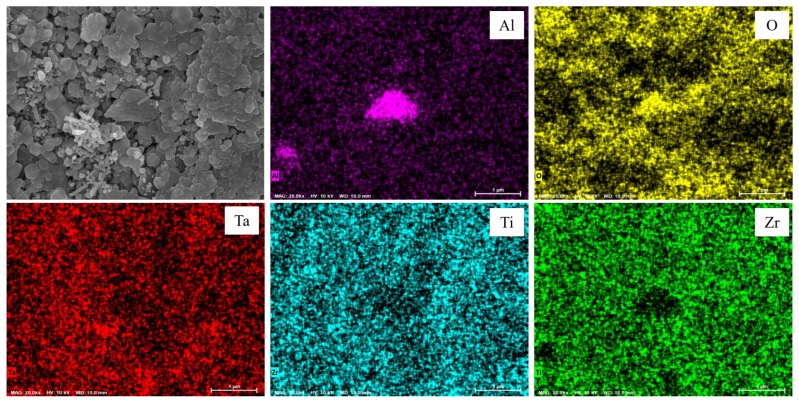
Elemental distribution maps of $Ti_{70}Zr_{20}Ta_{7}Al_{3}$ alloy.

**Figure 11:**
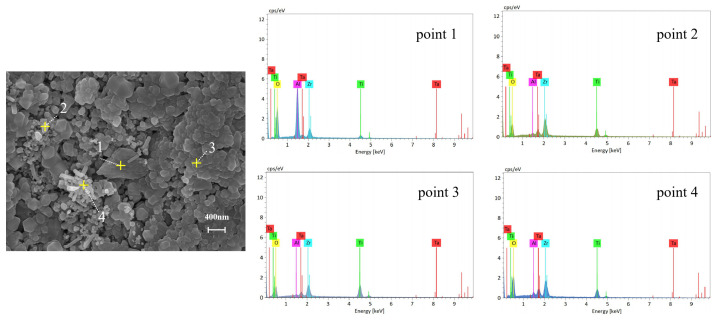
Elemental spectra at various points of $Ti_{70}Zr_{20}Ta_{7}Al_{3}$ alloy.

**Figure 12:**
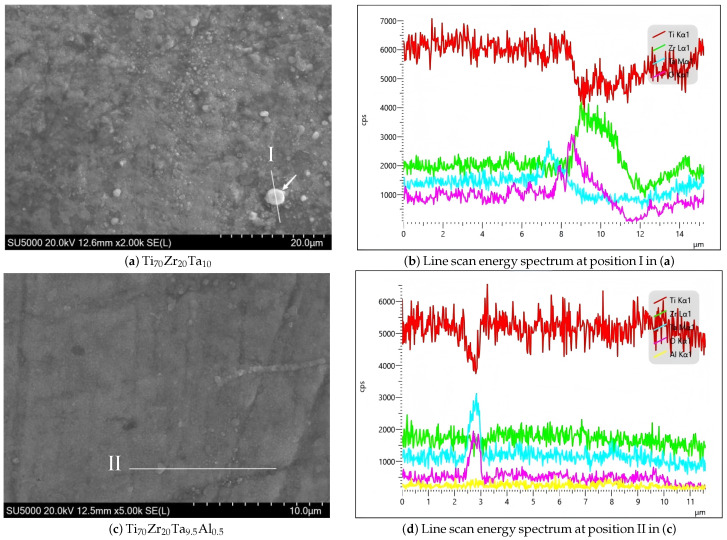
Surface morphology and line scan analysis of $Ti_{70}Zr_{20}Ta_{10}$ alloy and $Ti_{70}Zr_{20}Ta_{9.5}Al_{0.5}$ alloy.

Figure 10 and Figure 11 show the element distribution diagrams of $Ti_{70}Zr_{20}Ta_{9}Al_{1}$ alloy and $Ti_{70}Zr_{20}Ta_{7}Al_{3}$ alloy, respectively. Figure 12 shows the elemental analysis of $Ti_{70}Zr_{20}Ta_{7}Al_{3}$ alloy dots on the surface of the oxide layer. Table 3 shows the elemental content of points. As shown in Figure 9, when the Al content is 1 at.%, the oxide particles on the surface of the oxide film of $Ti_{70}Zr_{20}Ta_{9}Al_{1}$ alloy are uniformly and continuously distributed. Only in small areas do oxide particles exhibit intermittent phenomena. Meanwhile, Ti, Ta, Al, and O are uniformly distributed on the surface of $Ti_{70}Zr_{20}Ta_{9}Al_{1}$ alloy. As shown in Figure 10 and Figure 11, when the Al content is 3 at.%, a portion of Al undergoes segregation and forms block shaped particles. The main elements contained in the oxide particles are Al and O. Ta is mainly distributed in small sheet-like nanoparticles. The oxide film on the surface of the alloy is mainly composed of a mixed oxide of $\mathrm{Zr}O_{2}$ and $\mathrm{Ti}O_{2}$.

Figure 12 show the line scans of $Ti_{70}Zr_{20}Ta_{10}$ alloy and $Ti_{70}Zr_{20}Ta_{9.5}Al_{0.5}$ alloy, respectively. From Figure 12a,b, it can be seen that the content of Ti and Ta elements is very low in the white particles, while the content of Zr element is relatively high. Therefore, the white particles are probably $\mathrm{Zr}O_{2}$ phase. In the area indicated by the arrow in Figure 12a, the content of O element, Ta element and Ti element is relatively high, but there is almost no Zr element. Therefore, the phase in this region may be $Ta_{2}O_{5}$ phase. Figure 12d shows the line scan of $Ti_{70}Zr_{20}Ta_{9.5}Al_{0.5}$ alloy with an Al content of 0.5 at.%. As shown in the figure, the block shaped particles contain a relatively high amount of Ta and O elements, and almost no Ti element. Therefore, the phase in this region may be $Ta_{2}O_{5}$ phase.

In the initial stage of high-temperature oxidation of titanium alloys, chemical reactions occur rapidly. Outward-diffusing metal cations quickly combine with oxygen ions to form a loose and porous outer oxide film on the alloy surface, namely the near-surface oxide layer. After penetrating the surface oxide layer, oxygen ions react with metal cations under oxygen-deficient conditions to generate a dense inner oxide layer, defined as the diffusion layer. A small number of oxygen ions diffuse into the alloy matrix, forming the matrix oxidation layer. This layered oxide structure is a typical kinetic characteristic of diffusion-controlled oxidation [23,24].

**Figure 13:**
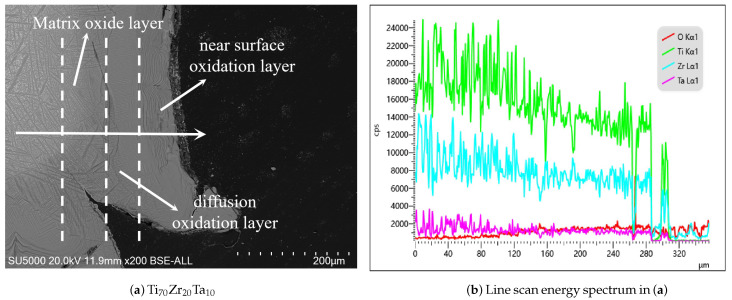
Cross-section and energy spectrum lines of $Ti_{70}Zr_{20}Ta_{10}$ alloy after oxidation at 900 °C for 10 h.

**Figure 14:**
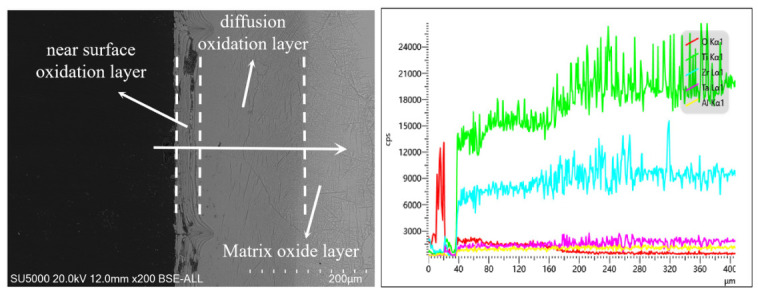
Cross-section and energy spectrum lines of ${\mathrm{Ti}}_{70}{\mathrm{Zr}}_{20}{\mathrm{Ta}}_{9.5}{\mathrm{Al}}_{0.5}{\mathrm{Al}}_{x}$ alloy after oxidation at 900 °C for 10 h.

**Figure 15:**
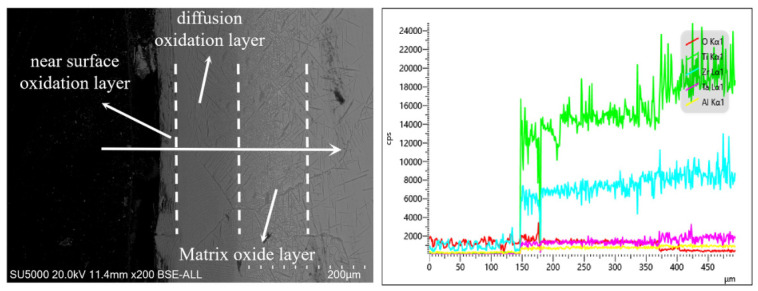
Cross-section and energy spectrum lines of $Ti_{70}Zr_{20}Ta_{9}Al_{1}$ alloy after oxidation at 900 °C for 10 h.

**Figure 16:**
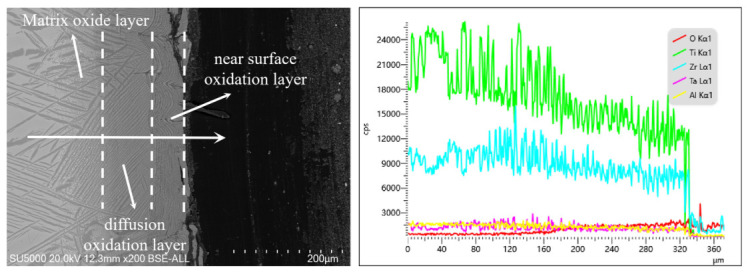
Cross-section and energy spectrum lines of $Ti_{70}Zr_{20}Ta_{7}Al_{3}$ alloy after oxidation at 900 °C for 10 h.

Figure 13, Figure 14, Figure 15 and Figure 16 show the cross-sectional morphology and corresponding energy spectrum scans of $Ti_{70}Zr_{20}Ta_{10-x}Al_{x}$ alloys with four different Al contents after isothermal oxidation at 900 °C for 10 h. As shown in Figure 13b, the oxide film of $Ti_{70}Zr_{20}Ta_{10}$ alloy is relatively loose. According to the contrast difference in Figure 13a, combined with the analysis of Figure 5 and Figure 13b, the oxide film on the surface of the alloy can be divided into three layers from the outside to the inside. The first layer of oxide film is relatively loose. The phase in this region is mainly composed of a large amount of $\mathrm{Ti}O_{2}$ and a small amount of $\left(\mathrm{Ti},\mathrm{Zr}\right)O_{2}$ phase. Its thickness is about 10 μm. In the second layer, the phase is mainly composed of the outer $Ta_{2}O_{5}$ phase and the inner mixed phase of $\mathrm{Ti}O_{2}$, $\left(\mathrm{Ti},\mathrm{Zr}\right)O_{2}$, and $Ta_{2}O_{5}$. Its thickness is about 190 μm. According to Figure 14, when the Al content is 0.5 at.%, there are pores and cracks in the alloy oxide film of the first layer. The oxide film on the second layer is relatively dense. The first layer of oxide film is mainly composed of $\mathrm{Ti}O_{2}$, $\mathrm{AlTiTa}O_{6}$, and $\left(\mathrm{Ti},\mathrm{Zr}\right)O_{2}$ phases. As shown in Figure 15, when the Al content is 1 at.%, the oxide film of the alloy can still be divided into three layers. The first layer of oxide film is not dense and contains pores and cracks. Compared with $Ti_{70}Zr_{20}Ta_{9.5}Al_{0.5}$, the number of pores in the oxide film of the first layer is relatively small. The thickness of the oxide film on the first and second layers is about 140 μm. The third layer is the diffusion layer, with a thickness of approximately 60 μm. According to Figure 16, when the Al content is 3 at.%, the oxide film of the alloy is similar to $Ti_{70}Zr_{20}Ta_{9.5}Al_{0.5}$. The thickness of the oxide film is about 200 μm. The thickness of the $Ti_{70}Zr_{20}Ta_{7}Al_{3}$ oxide film is smaller than that of $Ti_{70}Zr_{20}Ta_{9}Al_{1}$. 

**Figure 17:**
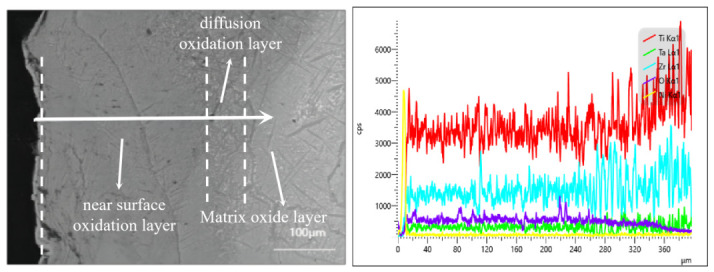
Cross-section and spectral lines of $Ti_{70}Zr_{20}Ta_{10}$ alloy after isothermal oxidation at 900 °C for 50 h.

**Figure 18:**
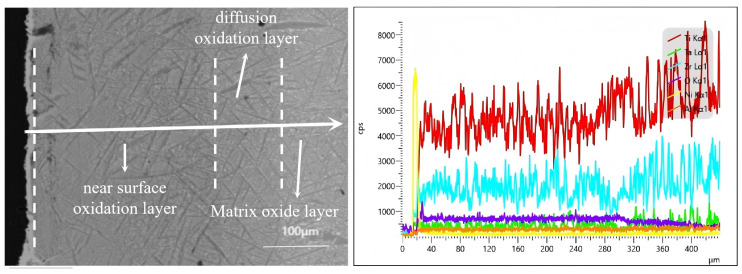
Cross-section and spectral lines of $Ti_{70}Zr_{20}Ta_{9.5}Al_{0.5}$ alloy after isothermal oxidation at 900 °C for 50 h.

**Figure 19:**
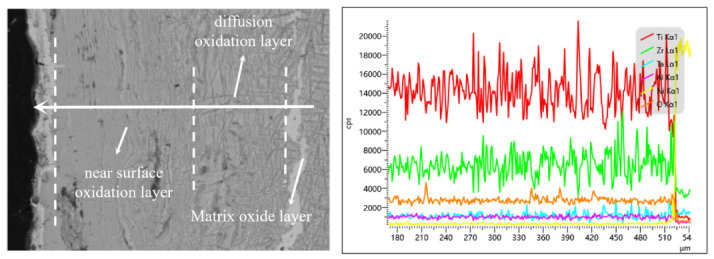
Cross-section and spectral lines of $Ti_{70}Zr_{20}Ta_{9}Al_{1}$ alloy after isothermal oxidation at 900 °C for 50 h.

**Figure 20:**
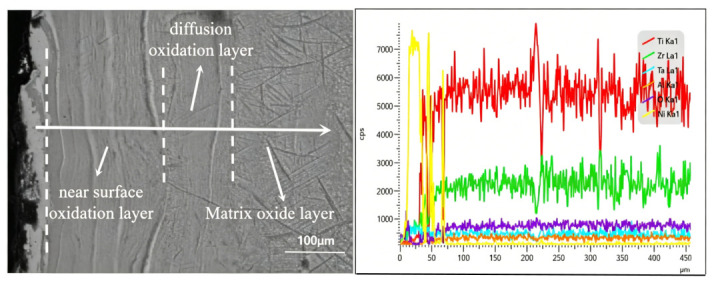
Cross-section and spectral lines of $Ti_{70}Zr_{20}Ta_{7}Al_{3}$ alloy after constant temperature oxidation at 900 °C for 50 h.

Figure 17, Figure 18, Figure 19 and Figure 20 show the cross-sectional morphology and energy spectrum scanning of the oxide film of four alloys after isothermal oxidation for 50 h. As shown in the figure, when the alloy is oxidized for 50 h, the thickness of the oxide film significantly increases compared to when the alloy is oxidized for 10 h. According to Figure 17, the oxide film of $Ti_{70}Zr_{20}Ta_{10}$ alloy is still in three layers. There are pores and cracks in the oxide film. And the closer to the surface of the oxide film, the more holes there are. This indicates that the oxide film is not very dense. Compared with the isothermal oxidation of $Ti_{70}Zr_{20}Ta_{10}$ alloy for 10 h, the thickness of the oxide film on the first and second layers increased significantly. The thickness of the oxide film is about 300 μm. When Al element is added to the alloy, the pores and cracks in the oxide film are reduced. The thickness of the oxide film on $Ti_{70}Zr_{20}Ta_{9.5}Al_{0.5}$ alloy is about 400 μm. The thickness of the oxide film on $Ti_{20}Zr_{20}Ta_{9}Al_{1}$ alloy and $Ti_{70}Zr_{20}Ta_{7}Al_{3}$ alloy is approximately 500 μm.

## 4. Conclusions

This paper systematically investigates the effect of Al addition on the high-temperature oxidation resistance of $Ti_{70}Zr_{20}Ta_{10-x}Al_{x}$ (x = 0, 0.5, 1, 3 at.%) alloys during isothermal oxidation at 900 °C. The oxidation kinetic constant (Kp) of the alloys was calculated via intermittent weighing. Subsequently, the phase composition and surface and cross-sectional micromorphology of the oxide layers were analyzed. The main conclusions are summarized as follows:

(1) The isothermal oxidation behavior of $Ti_{70}Zr_{20}Ta_{10-x}Al_{x}$ alloys at 900 °C fully follows the parabolic oxidation law. Among all tested alloys, the $Ti_{70}Zr_{20}Ta_{9}Al_{1}$ alloy exhibits the lowest Kp value, indicating that it possesses the slowest high-temperature oxidation reaction rate compared with the $Ti_{70}Zr_{20}Ta_{10}$, $Ti_{70}Zr_{20}Ta_{9.5}Al_{0.5}$, and $Ti_{70}Zr_{20}Ta_{7}Al_{3}$ alloys during isothermal oxidation at 900 °C.

(2) After isothermal oxidation at 900 °C, the oxide products formed on the surface of Al-containing $Ti_{70}Zr_{20}Ta_{10-x}Al_{x}$ (x = 0.5, 1, 3 at.%) alloys mainly include $\mathrm{Ti}O_{2}$, $Ta_{2}O_{5}$, $\left(\mathrm{Ti},\mathrm{Zr}\right)O_{2}$ and $\mathrm{AlTiTa}O_{6}$. By contrast, the oxidation products of the pure $Ti_{70}Zr_{20}Ta_{10}$ alloy primarily consist of $\mathrm{Ti}O_{2}$, $Ta_{2}O_{5}$ and $\left(\mathrm{Ti},\mathrm{Zr}\right)O_{2}$.

(3) Surface and cross-sectional characterization of the oxide layers confirms that the introduction of Al effectively improves the oxidation resistance of $Ti_{70}Zr_{20}Ta_{10}$ alloy. When the Al content increases to 3 at.%, obvious segregation of Al is observed in the oxide particles on the surface of $Ti_{70}Zr_{20}Ta_{7}Al_{3}$ alloy. In comparison, the $Ti_{70}Zr_{20}Ta_{9}Al_{1}$ alloy forms a denser and more integrated oxide layer. Consequently, the $Ti_{70}Zr_{20}Ta_{9}Al_{1}$ alloy exhibits superior high-temperature oxidation resistance among all experimental alloys.

## Figures and Tables

**Table 1:** $K_{P}$ values for each component at 900 °C for each time period.

*x* (at.%)	$K_{P}(\mathrm{mg}\cdot mm^{-4}\cdot h^{-1})$		
	0–5 h	15–30 h	30–50 h
0	0.12337±0.00253	0.08280±0.00346	0.08425±0.00547
0.5	0.11763±0.00571	0.07858±0.00473	0.08571±0.00723
1	0.10392±0.00492	0.06803±0.00526	0.07063±0.00775
3	0.12442±0.00672	0.07796±0.00457	0.07492±0.00680

**Table 2:** Elemental content of points in Figure 8b.

Position	Ti	Zr	Ta	Al	O
A	27.79	3.39	1.73	0.25	66.85
B	27.49	4.05	2.21	0.27	65.99

**Table 3:** Elemental content of points in Figure 11.

Position	Point 1	Point 2	Point 3	Point 4
Element	Norm.Atomic Concentration/%			
Oxygen	61.90	59.88	56.09	67.61
Titanium	5.86	24.54	34.03	20.17
Zirconium	4.28	12.48	7.77	8.86
Aluminium	27.47	1.56	1.12	1.90
Tantalum	0.50	1.54	0.99	1.46

## Data Availability

The original contributions presented in this study are included in the article. Further inquiries can be directed to the corresponding author.
